# Leisure-time physical activity, desire to increase physical activity, and mortality: A population-based prospective cohort study

**DOI:** 10.1016/j.pmedr.2023.102212

**Published:** 2023-04-23

**Authors:** Martin Lindström, Maria Rosvall, Mirnabi Pirouzifard

**Affiliations:** a Social Medicine and Health Policy, Department of Clinical Sciences and Centre for Primary Health Care Research, Lund University, S-205 02 Malmö, Sweden; b Department of Community Medicine and Public Health, Sahlgrenska Academy, Institute of Medicine, University of Gothenburg, Sweden

**Keywords:** Leisure-time physical activity, Physical activity promotion, Health-related behaviors, Mortality, Cardiovascular mortality, Cancer mortality, Sweden

## Abstract

The aim was to investigate associations between leisure-time physical activity (LTPA) and mortality, and associations between desire to increase LTPA and mortality within the low LTPA group. A public health survey questionnaire was sent in 2008 to a stratified random sample of the population aged 18–80 in southernmost Sweden, yielding a 54.1% response rate. Baseline 2008 survey data with 25,464 respondents was linked to cause of death register data to create a prospective cohort with 8.3-year follow-up. Associations between LTPA, desire to increase LTPA and mortality were analyzed in logistic regression models. An 18.4% proportion performed regular exercise (at least 90 min/week, leading to sweating), 23.2% moderate regular exercise (once or twice a week at least 30 min/occasion, leading to sweating), 44.3% moderate exercise (more than two hours walking or equivalent activity/week) and 14.1% reported low LTPA (less than two hours walking or equivalent activity/week). These four LTPA groups were significantly associated with covariates included in the multiple analyses. The results showed significantly higher all-cause, cardiovascular (CVD**)**, cancer and other cause mortality for the low LTPA group but not for the moderate regular exercise and moderate exercise groups compared to the regular exercise group. Both the “Yes, but I need support” and the “No” fractions within the low LTPA group had significantly increased ORs of all-cause mortality compared to the “Yes, and I can do it myself” reference, while no significant associations were observed for CVD mortality. Physical activity promotion is particularly warranted in the low LTPA group.

## 1 Introduction

Physical activity promotes health and prevents a number of prevalent chronic diseases and their progression over the life course. Physical activity is associated with all-cause mortality, cardiovascular (CVD) mortality and mortality from a number of cancer diagnoses (Mora et al., 2007, Lee et al., 2012, Moore et al., 2016, Zhao et al., 2020). It is also associated with increasingly improved health and reduced mortality even above lowest recommended levels of physical activity in a stepwise association (McTiernan et al., 2019). Recent studies suggest that middle-aged and older adults, including those with CVD and cancer diagnoses, can increase survival by becoming more physically active, regardless of earlier physical activity levels in the life trajectory, and regardless of already established risk factors (Mok et al., 2019).

Current evidence-based recommendations from 2018 suggest that for substantial health benefits, adults should do at least 75–150 min/week vigorous intensity aerobic or 150–300 min/week moderate intensity physical activity, or a comparable combination of vigorous and moderate intensity physical activity. Preferably, vigorous activity should be distributed over the week (Physical Activity Guidelines Advisory Committee, 2018). The current literature suggests that there are health protective effects to gain from increased physical activity well beyond the minimum recommended levels of physical activity. In one recent study, a substantial reduction of mortality risk was observed between the group spending 500–999 kcal on physical activity per week compared to the inactive physical activity group spending less than 500 kcal per week. There was a graded dose–response association, which entailed decreasing mortality until approximately 3000 kcal expenditure on physical activity per week, beyond which no further mortality reduction was observed. Energy expenditure from vigorous physical activity seemed to have a stronger protective effect than the total amount of physical activity (Ekelund et al., 2020). A higher proportion of vigorous physical activity (VPA) of all physical activity also seems to be associated with lower all-cause mortality (Wang et al., 2021). In another study, high levels of moderate intensity physical activity, i.e. 60–75 min per day, seemed to eliminate excess mortality associated with sedentary (long sitting) time. The same study suggests that more time spent on physical activity at any level of intensity is associated with substantially reduced mortality risk in middle-aged and older adults (Ekelund et al., 2016, Ekelund et al., 2019).

Many patients visiting general practitioners are physically inactive, which makes general practice one of several important contact surfaces for physical activity promotion. In general practice, physical activity should be promoted for health gain, particularly for patients reporting poor health (Falskog et al., 2021). Many men and women with low leisure-time physical activity (LTPA) report a desire to increase physical activity, but also report a need for support to do so (Lindström, 2011). Help and guidance from healthcare professionals may be needed. Physical activity counsellors in general are also important for sustained physical activity for reasons of their individual-oriented approach (Andersen et al., 2019).

A cross-sectional population-based study conducted in the adult population in southern Sweden in 2004 showed that 30.7% of the population answered that they had “no” desire to increase physical activity, 51.0% answered “yes, and I can do it myself”, and 18.3% answered “yes, but I need support”. (Lindström, 2011). To our knowledge, no study has previously investigated the associations between self-reported desire to increase physical activity at baseline and all-cause, CVD, cancer and other cause mortality among respondents with low LTPA at baseline longitudinally.

The aim of this study is to investigate associations between LTPA and all-cause, CVD, cancer and other cause mortality, adjusting for relevant covariates. A second aim is to analyze associations between desire to increase LTPA and mortality within the low LTPA group, adjusting for relevant covariates.

## 2 Material and methods

### 2.1 Study population

In the autumn of 2008 a public health survey was conducted in Scania, the southernmost part of Sweden. The survey was cross-sectional and based on a stratified sample of the adult register population aged 18–80. A postal questionnaire was sent. There were also three postal reminders. The questionnaire could also be completed online. A sum of 28,198 responded, which yielded a 54.1% participation rate. The public health survey was conducted by *Region Skåne*, which is the regional public authority responsible for the healthcare system in Scania. The questionnaire included 134 questions regarding sociodemographic, self-rated general health, self-rated psychological health, trust, social participation, social support, health-related behaviors and items related to crime. The random sample was stratified geographically based on 29 smaller municipalities and 30 city parts in the four major cities (Malmö, Lund, Helsingborg and Kristianstad). The number of participants in this geographical stratification was based on age, sex and education to achieve statistical power in all approximately 60 municipalities/city parts. The stratified sample was drawn from the public register population in cooperation with *Statistics Sweden*. *Statistics Sweden* also created the population weight, which compensates for the stratification in order to achieve representativeness of the entire Scania population. The 2008 cross-sectional public health survey baseline data was linked to register-based cause of death data (*Dödsorsaksregistret*) obtained from the *National Board of Health and Welfare* (*Socialstyrelsen*), which yielded a closed prospective cohort population.

This study was approved by the Ethical Committee (*Etikprövningsnämnden*) in Lund (No. 2010/343).

### 2.2 Dependent variable

All-cause and diagnosis-specific mortality was followed prospectively from 27 August-14 November 2008 (exact date according to registration date of individual answers) until 31 December 2016, i.e. approximately 8.3 years later, or until death. In total, 25,464 participants were included in the present study (11,589 men and 13,875 women), while 2,462 respondents with internally missing values on any or several of the variables analyzed in this study were excluded. An additional 136 respondents in 2008 were lost to follow-up. ICD 10 (International Classification of Diseases 10) was used for causes of death (*Dödsorsaksregistret*). The Swedish ten-digit person number system makes possible the linkage of baseline public health questionnaire survey data with the national cause of death register at the *National Board on Health and Welfare* (*Socialstyrelsen*). The linkage was performed through a third party (private company). The ten-digit person numbers were erased from the data set before delivery to the research group.

All-cause (total), cardiovascular (CVD) (I00-I98), cancer (C00-C97), and all other causes (other causes than I00-I98 and C00-C97) mortality were analyzed in this study. All-cause mortality is the sum of the three cause-specific groups.

### 2.3 Independent variables

*Leisure-time physical activity* (LTPA) was obtained with the four alternatives regular exercise (at least three times per week at least 30 min/ occasion, leading to sweating), moderate regular exercise (exercising once or twice per week at least 30 min/ occasion, leading to sweating), moderate exercise (more than 2 h walking, cycling or equivalent activity/ week) and low LTPA (less than 2 h walking, cycling or equivalent activity/ week). The LTPA variable has been defined previously (Lindström and Rosvall, 2018).

*Desire to increase physical activity* was assessed with the item “Do you want to increase your physical activity?”, with the alternative answers “Yes, and I think I can do it myself”, “Yes, but I need support”, and “No”.

Men and women were collapsed throughout the analyses.

*Age* was analyzed as a continuous variable in the models.

*Country of birth* was defined as either born in Sweden or born abroad.

*Socioeconomic status (SES)* (by occupation and standing on the labor market) was defined into non-manual employees in higher, medium and lower occupational positions, skilled and unskilled manual workers, and self-employed. The categories outside the workforce were defined as unemployed (job seekers), early retired (below 65), old-age pensioners (65 and above), students, long-term sick leave, and unclassified.

*Chronic disease* was measured with the item “Do you have any long-term disease, ailment or injury, any disability or other weakness?”, with the alternative answers “Yes” and “No”.

*Body mass index (BMI)* was self-reported by weight (kg) and height (m) in the public health survey in 2008. BMI was analyzed as a continuous variable.

*Tobacco smoking* was measured with the item “Do you smoke?” with the alternative answers daily, non-daily and non-smoker. The two latter alternatives were collapsed, yielding “daily smoker” as risk group.

*Alcohol consumption during the past year* was measured with the item “How often have you consumed alcohol during the past 12 months?”, with the alternative answers “daily or almost daily”, “several occasions per week”, “once per week”, “2–3 times per month”, “once per month”, “once or a few times per half year”, or “more seldom or never”.

### 2.4 Statistics

**Fig. 1:**
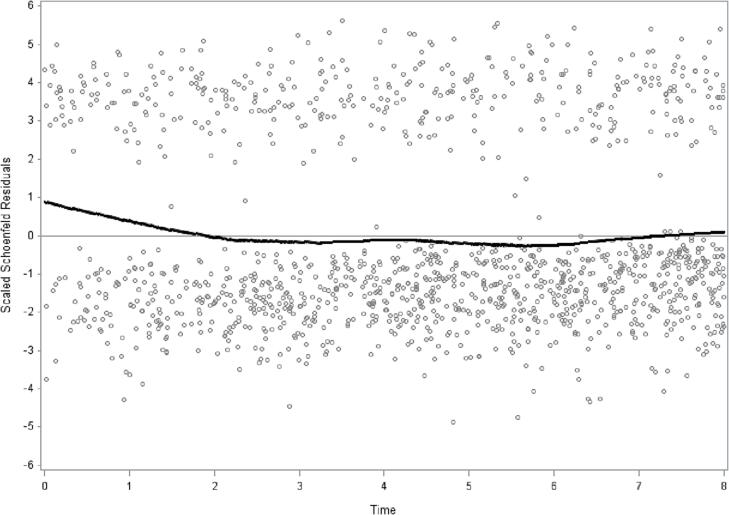
Schoenfeld residuals according to all-cause mortality and leisure-time physical activity (LTPA) (by placing the groups regular exercise, moderate regular exercise and moderate exercise in one group, and the low LTPA group in the other) over the 8.3-year period. The proportionality test with interaction term between LTPA and all-cause mortality across the 8.3-year period is significant, p = 0.005, which indicates non-proportionality. The 2008 public health survey in Scania with 8.3 years follow-up. Men and women combined. n = 25 464.

Prevalence (%) of all variables stratified by the four alternatives of the LTPA item were calculated. The differences between the LTPA alternatives were calculated with *t*-test for continuous variables and chi-square test for categorical variables (p-values) (Table 1). The assumption of proportional hazards was determined by introducing an interaction term with time, LTPA and all-cause mortality to test the assumption of proportional hazards. Schoenfeld residuals were assessed for LTPA and all-cause mortality (Kleinbaum and Klein, 1996, Kuitunen et al., 2021). The proportionality test with interaction term between LTPA and all-cause mortality across the 8.3-year period was significant, p = 0.005, indicating non-proportionality. Schoenfeld residuals**,** with low LTPA compared to the aggregate of the other three categories with regard to all-cause mortality**,** also indicated non-proportionality (Fig. 1). The observed non-proportionality is the reason why logistic regression models with odds ratios were applied in this study. Odds ratios (ORs) with 95% confidence intervals (95% CI:s) of all-cause, CVD, cancer and other cause mortality by the four LTPA alternatives were calculated. Four models were calculated: model 0 unadjusted, model 1 adjusted for sex and age, model 2 adjusted for sex, age, country of birth, SES, and chronic disease, model 3 additionally adjusted for BMI, smoking and alcohol consumption (Table 2). Odds ratios (ORs) with 95% confidence intervals (95% CI:s) of all-cause, CVD, cancer and other cause mortality in the low LTPA category were calculated according to the three desire to increase physical activity alternatives, with “Yes, and I think that I can do it myself” as reference category. Four models were calculated: unadjusted (model 0), adjusted for sex and age (model 1), additionally adjusted for country of birth, SES, and chronic disease (model 2), and additionally adjusted for BMI, smoking and alcohol consumption (model 3) (Table 3). Follow-up days were counted from baseline to death or last follow-up date (31 December 2016). Analysis of sampling variability without distributional assumptions applied to the study population is possible by bootstrap analysis (SAS/STAT Software Survey Analysis, 2021). Accurate variance estimation on weighted data with confidence intervals and p-values were achieved by bootstrap analyses with 1000 replications. The SAS software version 9.4 was used for calculations.

**Table 1:** Descriptive characteristics (%) of the participants by level of leisure-time physical activity (LTPA) by four levels of leisure-time physical activity (LTPA). The 2008 public health survey in Scania, Sweden. Total population n = 25464. Weighted prevalence.

		Frequency of leisure-time physical activity (LTPA).				
	All	Regular exercise	Moderate regular exercise	Moderate exercise	Low LTPA	p-value
	n = 25464	n = 4558	n = 5950	n = 11660	n = 3296	
		18.4%	23.2%	44.3%	14.1%	
Deaths (n)	1365	157	179	632	397	
Age, yrs: mean ± SD a	46.1 ± 16.9 (45.9–46.4)	41.8 ± 17.4 (41.1–42.4)	43.5 ± 15.8 (43.0–44.0)	49.0 ± 16.4 (48.6–49.4)	47.1 ± 17.3 (46.4–47.8)	<0.001
BMI: mean ± SD a	25.6 ± 4.5 (25.5–25.6)	24.4 ± 3.6 (24.3–24.6)	25.2 ± 4.2 (25.0–25.3)	25.7 ± 4.4 (25.6–25.8)	27.1 ± 5.8 (26.9–27.4)	<0.001

Sex^b^						
Male	50.2 (49.4–51.0)	53.1 (51.2–54.9)	52.0 (50.4–53.5)	46.5 (45.3–47.6)	55.2 (53.1–57.3)	<0.001
Female	49.8 (49.0–50.6)	46.9 (45.1–48.8)	48.0 (46.5–49.6)	53.5 (52.4–54.7)	44.8 (42.7–46.9)	

Socioeconomic status (SES) b						<0.001
Higher non-manual	8.9 (8.5–9.3)	10.8 (9.6–12.0)	12.0 (11.0–13.0)	7.5 (7.0–8.1)	5.3 (4.5–6.2)	
Medium non-manual	13.8 (8.5–14.3)	14.5 (13.3–15.7)	18.4 (17.2–16.7)	12.8 (12.1–13.5)	8.4 (7.2–9.5)	
Lower non-manual	7.9 (7.5–8.3)	8.1 (7.1–9.1)	8.6 (7.8–9.5)	7.9 (7.2–8.5)	6.6 (5.6–7.6)	
Skilled manual	10.5 (10.0–11.0)	10.3 (9.1–11.4)	10.3 (9.3–11.3)	11.0 (10.3–11.7)	9.4 (8.1–10.7)	
Unskilled manual	12.5 (11.9–13.0)	11.4 (10.1–12.6)	10.7 (9.7–11.7)	13.2 (12.4–14.0)	14.6 (13.0–16.2)	
Self-employed/farmer	6.0 (5.6–6.4)	5.3 (4.5–6.1)	5.5 (4.8–6.2)	6.4 (5.8–6.9)	6.7 (5.5–7.8)	
Early retired	3.9 (3.6–4.2)	2.6 (2.1–3.1)	2.3 (1.8–2.8)	3.9 (3.5–4.4)	8.2 (7.0–9.4)	
Unemployed	4.0 (3.7–4.3)	3.8 (3.1–4.6)	3.0 (2.4–3.6)	3.6 (3.1–4.1)	7.1 (5.9–8.3)	
Student	8.3 (7.8–8.7)	11.7 (10.4–13.0)	9.6 (8.5–10.6)	6.5 (5.8–7.1)	7.4 (6.1–8.7)	
Old age pensioner	17.7 (17.2–18.3)	14.2 (13.2–15.3)	13.3 (12.4–14.3)	21.6 (20.7–22.5)	17.4 (16.0–18.9)	
Unclassified	5.4 (5.0–5.8)	6.8 (5.7–7.8)	5.4 (4.6–6.3)	4.4 (3.9–5.0)	6.4 (5.1–7.7)	
Long-term sickleave	1.1 (1.0–1.3)	0.6 (0.3–0.8)	0.7 (0.4–1.0)	1.2 (0.9–1.4)	2.5 (1.9–3.2)	
Born outside Sweden (ref. born in Sweden)^b^	18.1 (17.4–18.7)	15.0 (13.5–16.4)	15.1 (13.7–16.4)	17.1 (16.1–18.1)	30.0 (27.8–32.2)	<0.001
Chronic disease (ref. no chronic disease)^b^	28.6 (27.9–29.3)	22.4 (21.0–23.9)	23.3 (22.0–24.7)	29.9 (28.9–30.9)	41.0 (38.9–43.1)	<0.001
Daily smoking (ref. non-daily + non smoker)^b^	14.5 (14.0–15.1)	7.9 (6.8–8.9)	9.8 (8.8–10.8)	16.2 (15.3–17.1)	25.7 (23.8–27.7)	<0.001

Alcohol drinking past year^b^						<0.001
Never	11.9 (11.4–12.4)	9.1 (7.9–10.2)	7.4 (6.5–8.3)	12.5 (11.7–13.3)	21.0 (19.1–22.8)	
Once a month or more seldom	22.9 (22.2–23.5)	21.6 (20.1–23.1)	19.7 (18.4–21.0)	23.3 (22.4–24.3)	28.4 (26.4–30.3)	
2–4 times a month	35.6 (34.9–36.3)	39.7 (37.9–41.6)	39.5 (37.9–41.1)	34.5 (33.4–35.7)	27.2 (25.1–29.2)	
2–3 times a week	22.4 (21.8–23.1)	23.2 (21.8–24.7)	27.0 (25.6–28.4)	22.2 (21.2–23.2)	14.7 (13.2–16.2)	
At least 4 times a week	7.2 (6.8–7.6)	6.4 (5.6–7.2)	6.4 (5.7–7.1)	7.4 (6.9–8.0)	8.8 (7.6–10.0)	

Desire to increase physical activity						
“Yes, and I can do it myself”	54.4 (53.6–55.2)	49.4 (47.6–51.2)	66.7 (65.2–68.2)	55.7 (54.6–56.7)	36.8 (34.7–38.9)	<0.001
“Yes, but I need support”	20.2 (19.6–20.8)	6.0 (5.1–6.9)	12.4 (11.3–13.5)	22.9 (21.9–23.8)	43.1 (40.9–45.3)	
“No”	25.4 (24.7–26.1)	44.6 (42.8–46.3)	21.0 (19.7–22.2)	21.5 (20.6–22.4)	20.1 (18.3–21.9)	

The values in parentheses are 95% confidence intervals for mean or percent based on bootstrap method with 1000 number of replicates.

^a^ p-value: Independent samples ANOVA-test, 2-tailed.

b p-value: Pearson Chi Square test, 2-sided.

**Table 2:** Odds ratios (ORs) with 95% confidence intervals (95% CIs) of all-cause, cardiovascular (CVD), cancer and other cause mortality according to leisure-time physical activity (LTPA). The 2008 public health survey in Scania with 8.3 years follow-up. Men and women combined. Total population n = 25 464. Weighted prevalence.

	Model 0		Model 1		Model 2		Model 3		
Cause of death	OR	(95 %CI)	OR	(95 %CI)	OR	(95 %CI)	OR	(95 %CI)	Number of deaths
All cause mortality									1365
Regular exercise	1.0		1.0		1.0		1.0		
Moderate regular exercise	0.8	(0.6–1.1)	0.8	(0.6–1.1)	0.9	(0.6–1.1)	0.8	(0.6–1.1)	
Moderate exercise	1.8***	(1.5–2.2)	1.1	(0.9–1.4)	1.1	(0.9–1.4)	1.1	(0.8–1.3)	
Low LTPA	3.9***	(3.1–4.9)	3.5***	(2.8–4.4)	2.9***	(2.3–3.7)	2.5***	(2.0–3.3)	

CVD mortality									416
Regular exercise	1.0		1.0		1.0		1.0		
Moderate regular exercise	0.8	(0.5–1.2)	0.8	(0.5–1.3)	0.8	(0.5–1.3)	0.8	(0.5–1.2)	
Moderate exercise	1.6*	(1.1–2.3)	1.0	(0.7–1.5)	1.0	(0.7–1.4)	0.9	(0.6–1.3)	
Low LTPA	3.6***	(2.4–5.3)	2.7***	(1.8–4.1)	2.2***	(1.5–3.4)	1.7*	(1.1–2.7)	

Cancer mortality									528
Regular exercise	1.0		1.0		1.0		1.0		
Moderate regular exercise	0.9	(0.6–1.4)	0.9	(0.6–1.4)	0.9	(0.6–1.4)	0.9	(0.6–1.4)	
Moderate exercise	2.0***	(1.4–2.9)	1.3	(0.9–1.9)	1.3	(0.9–1.8)	1.3	(0.9–1.8)	
Low LTPA	2.9***	(2.0–4.2)	2.1***	(1.4–3.1)	1.9***	(1.3–2.8)	1.7**	(1.2–2.5)	

Other cause mortality									421
Regular exercise	1.0		1.0		1.0		1.0		
Moderate regular exercise	0.8	(0.5–1.4)	0.9	(0.5–1.4)	0.9	(0.5–1.4)	0.9	(0.5–1.4)	
Moderate exercise	1.7**	(1.2–2.5)	1.1	(0.7–1.6)	1.1	(0.7–1.6)	1.0	(0.7–1.5)	
Low LTPA	5.0***	(3.4–7.4)	3.9***	(2.6–5.8)	3.1***	(2.1–4.7)	3.0***	(1.9–4.6)	

Model 0 unadjusted. Model 1 adjusted for sex and age. Model 2 additionally adjusted for socioeconomic status, country of birth and chronic disease. Model 3 additionally adjusted for BMI, daily smoking and alcohol consumption.

Significance levels: * p < 0.05, ** p < 0.01, *** p <n 0.001; Weighted Hazard Ratios. Bootstrap method (1000 replicates) for variation estimation.

**Table 3:** Odds ratios (ORs) with 95% confidence intervals (95% CIs) of all-cause, cardiovascular (CVD), cancer and other cause mortality according to desire to increase physical activity in the low LTPA group. The 2008 public health survey in Scania with 8.3 years follow-up. Men and women combined. Total population **n=3 296**. **Weighted prevalence.**

	**Model 0**		**Model 1**		**Model 2**		**Model 3**		
**Cause of death**	**OR**	**(95%CI)**	**OR**	**(95%CI)**	**OR**	**(95%CI)**	**OR**	**(95%CI)**	**Number of deaths**
**All cause mortality**									**397**
“Yes, and I can do it myself”	1.0		1.0		1.0		1.0		
“Yes, but I need support”	2.3***	(1.7-3.2)	2.0***	(1.4-2.9)	1.6*	(1.0-2.3)	1.6*	(1.0-2.4)	
“No”	3.8***	(2.6-5.5)	2.0***	(1.3-3.0)	1.7**	(1.2-2.6)	1.7*	(1.1-2.7)	

**CVD mortality**									**128**
“Yes, and I can do it myself”	1.0		1.0		1.0		1.0		
“Yes, but I need support”	1.4	(0.8-2.5)	1.0	(0.6-1.9)	0.9	(0.5-1.6)	0.9	(0.5-1.7)	
“No”	2.7**	(1.5-4.9)	1.2	(0.7-2.4)	1.1	(0.6-2.2)	1.2	(0.6-2.3)	

**Cancer mortality**									**110**
“Yes, and I can do it myself”	1.0		1.0		1.0		1.0		
“Yes, but I need support”	1.9*	(1.0-3.4)	1.4	(0.8-2.6)	1.1	(0.6-2.1)	1.1	(0.6-2.2)	
“No”	4.0***	(2.1-7.5)	2.2*	(1.1-4.3)	1.9*	(1.0-3.7)	2.0*	(1.0-3.9)	

**Other cause mortality**									**159**
”Yes, and I can do it myself”	1.0		1.0		1.0		1.0		
“Yes, but I need support”	3.8***	(2.1-6.9)	3.0***	(1.6-5.6)	2.5**	(1.3-4.8)	2.4*	(1.2-4.9)	
“No”	4.0***	(2.2-7.4)	1.9*	(1.0-3.5)	1.7	(0.9-3.1)	1.6	(0.8-3.0)	

Model 0 unadjusted. Model 1 adjusted for sex and age. Model 2 additionally adjusted for socioeconomic status, country of birth and chronic disease. Model 3 additionally adjusted for BMI, daily smoking and alcohol consumption.

Significance levels: * p<0.05, ** p<0.01, *** p<0.001; Weighted Hazard Ratios. Bootstrap method (1000 replicates) for variation estimation.

## 3 Results

Table 1 shows that 18.4% of respondents performed regular exercise, 23.2% moderate regular exercise, 44.3% moderate exercise and 14.1% reported low LTPA. The proportions with self-reporting chronic disease were significantly higher in the moderate exercise and low LTPA groups compared to both the regular exercise and moderate regular exercise groups. The prevalence of daily smoking was 7.9% (6.8%-8.9%) among respondents reporting regular exercise, 9.8% (8.8%-10.8%) among respondents reporting moderate regular exercise, 16.2% (15.3%-17.1%) among respondents reporting moderate exercise and 25.7% (23.8%-27.7%) among respondents reporting low LTPA. Respondents in the low LTPA group reported that they need support to increase physical activity to a significantly higher extent, 43.1% (40.9%-45.3%), than in any of the other three LTPA groups: regular exercise, 6.0% (5.1%-6.9%), moderate regular exercise, 12.4% (11.3%-13.5%), and moderate exercise, 21.9% (20.9%–23.1%), groups. All items are displayed in Table 1.

Table 2 shows that both the moderate exercise and low LTPA groups display significantly higher ORs than the regular exercise reference group in the unadjusted logistic regression analyses for all-cause, CVD, cancer and other cause mortality in model 0. After adjustments for sex and age in model 1, all ORs for all-cause, CVD, cancer and other cause mortality for the moderate exercise group became not significant and close to the reference regular exercise group, OR 1.00. In contrast, the ORs for the low LTPA group remained statistically significant with high ORs (effect measures) throughout the multiple analyses, with the low LTPA group showing OR 2.5 (2.0–3.3) for all-cause mortality, OR 1.7 (1.1–2.7) for CVD mortality, OR 1.7 (1.2–2.5) for cancer mortality, and OR 3.0 (1.9–4.6) for other cause mortality in the final fully adjusted model 3.

Table 3 shows results regarding desire to increase LTPA in the low LTPA group. Both the “Yes, but I need support” and the “No” fractions had significantly increased ORs of all-cause mortality throughout the multiple analyses in models 0–3 compared to the reference (“Yes, I have desire to increase my LTPA and I can do it myself”) category. No statistically significant differences in CVD mortality were observed in models 1–3, with all effect measures (ORs) close to 1.00. In contrast, the part of the low LTPA group with no desire to increase LTPA displayed significantly higher ORs of cancer mortality in models 1–3. The “Yes, but I need support to increase LTPA” fraction of the low LTPA group displayed significantly higher other cause mortality in models 1–3. Results from survival (Cox) regression analyses very similar to the results in Table 2, Table 3 are displayed in supplementary tables S1 and S2. Table S3 displays results from logistic regression analyses with the two middle alternatives of the LTPA item collapsed.

## 4 Discussion

The associations between LTPA and mortality showed significantly higher all-cause, CVD, cancer and other cause mortality for the low LTPA group compared to the regular exercise reference group throughout the multiple analyses, but not for the moderate regular exercise and moderate exercise groups. Regarding desire to increase LTPA, both the “Yes, but I need support” and the “No” fractions within the low LTPA group had significantly increased ORs of all-cause mortality throughout the multiple logistic regression analyses compared to the “Yes, and I can do it myself” reference. No significant associations were observed for CVD mortality with regard to desire to increase physical activity in the low LTPA group. The “no” desire to increase LTPA fraction displayed significantly higher cancer mortality, and the “Yes, but I need support” fraction had significantly higher other cause mortality throughout models 0–3.

The low LTPA group was defined somewhat lower than the lowest recommended level of LTPA in the present study. As would be expected from the literature, the low LTPA group displayed significantly higher all-cause, CVD, cancer and other cause mortality compared to the regular exercise reference group. In contrast to expectations, no significantly higher all-cause, CVD, cancer and other cause mortality was observed for the moderate regular and moderate exercise groups compared to the regular exercise reference group. Such patterns would have been expected, given the fact that the recent literature suggests increased health benefits and a lowering effect on mortality of increased physical activity at levels well above lowest recommended levels of exercise (Ekelund et al., 2016, Ekelund et al., 2019, Ekelund et al., 2020, Wang et al., 2021). Still, the two middle alternatives of the LTPA item do not include combinations of vigorous and moderate intensity LTPA following the 2018 guidelines.

A recent systematic review and *meta*-analysis of objective measures of physical activity and all-cause mortality found that current recommendations for LTPA based on subjective measurement from questionnaire items may underestimate the true reduction in mortality risk from increased physical activity and LTPA (Ramakrishnan et al., 2021). The results in Table 2 in the present study, which show no significantly increased ORs of mortality in the moderate regular and moderate exercise groups, may be an illustration of the conclusions drawn in that systematic review.

A comparatively high proportion of respondents within the low LTPA group report a desire to increase physical activity in combination with a need for support to meet this desire. Both this fraction of the low LTPA group and the fraction of the low LTPA group that reports no desire to increase physical activity have significantly higher all-cause mortality. There thus seems to be a substantial fraction within the low LTPA group that would be susceptible to interventions from healthcare professionals, and other forms of physical activity promotion (Arem et al., 2015, Falskog et al., 2021, Lindström, 2011). Physical activity on prescription may be one way to increase LTPA in this low LTPA group (Andersen et al., 2019), but other measures tailored to individual preconditions should also be practiced**.** The promotion of physical activity should also include professionals from the sport sector and physiotherapy to encourage and promote ideas how to be physically active.

The 14% prevalence of low LTPA is similar to the prevalence levels observed in other public health surveys conducted by *Region Skåne* in Scania between 2000 and 2019. The same prevalence of low LTPA is also observed in national public health surveys in Sweden conducted by the Public Health Agency (*Folkhälsomyndigheten*) (Heimerson, 2014) and other European studies, e.g. the 12.4% (295/2383) prevalence of “inactive” among men and 15.2% (362/2389) among women in the Dutch GLOBE study (Groeniger et al., 2017).

The SES patterns of daily smoking displayed in this study are well known in Sweden and other high-income countries. In contrast, alcohol consumption in Sweden displays a weak reverse SES gradient and a tendency towards slightly higher alcohol consumption at the group level in higher SES groups. Additionally, complete alcohol abstention is much more common in lower SES groups than in higher for historical reasons including the temperance movement (Lindström, 2005). The alcohol consumption item reflects the Nordic European pattern of more clustered and intense consumption normally concentrated at weekends. In contrast, the Mediterranean pattern entails small or moderate alcohol consumption, often wine, almost every day (Castelli et al., 2022).

The 8.3-year follow-up period displays a lack of proportionality between low LTPA and all-cause mortality**,** with a stronger association over the first years compared to the later part of the 8.3-year period, which is the reason for the use of logistic regression analyses instead of survival (Cox) regression analyses. Still, survival (Cox) regression analyses applied to the 8.3-year follow-up period show the same patterns and significant results as those displayed in Table 2, Table 3 (see supplementary tables S1 and S2). The optimal study design would have been panel data design with at least three observation points for LTPA, desire to increase physical activity and the other questionnaire variables. However, such a panel data design is not available in our data.

The self-reported chronic disease item was introduced in models 2–3 as an approximation of health at baseline, and an adjustment for health at baseline.

## 5 Strengths and limitations

This study is large, population-based and longitudinal, although the low LTPA group (n = 3,296, including 397 deaths) analyzed in Table 3 is comparatively small. The respondent population in the 2008 baseline public health survey is sufficiently representative in terms of age, sex and education of the general parameter population aged 18–80 in Scania in 2008. Age-specific mortality in the age groups 18–44, 45–64 and 65–80 was also comparable to Swedish age-specific mortality in the same age groups. The risk of selection bias is limited (Lindström and Rosvall, 2019).

The LTPA item includes four different levels of LTPA. Current recommendations are related to heart beats and breathing rather than sweating (Lindström, 2011). Still, the effective recommendation to exercise at least 150 min per week remains, which means that less than 2 h (less than 120 min) per week of moderate exercise is somewhat lower than the recommendation (Physical Activity Guidelines Advisory Committee, 2018). The two middle alternatives moderate regular and moderate exercise of the LTPA item do not include combinations of vigorous and moderate intensity LTPA. They are thus not fully compatible with the 2018 physical activity recommendations. The validity and reproducibility of LTPA questionnaire items with four alternatives are acceptable compared to golden standards assessing heart rate monitoring, which have been evaluated against whole-body calorimetry and double-labelled water (Wareham et al., 2003). Swedish register data including data regarding causes of death are generally regarded as valid, although the current low autopsy rates have probably led to somewhat decreased validity. The cancer diagnoses probably have highest validity as causes of death, but the broadness of the three groups of diagnoses included in this study ensures high validity between these three groups. The item regarding chronic disease was included to adjust for subjective and self-perceived health problems present at baseline. Men and women were analyzed together due to concerns regarding statistical power, particularly in Table 3. This strategy is supported by the fact that the prevalence of LTPA is similar for men and women, and that the health outcomes of different levels of LTPA are similar.

Relevant confounders including age, country of birth, SES, baseline chronic disease, BMI, tobacco smoking and alcohol consumption were included in the multiple logistic regression analyses.

## 6 Conclusion

The associations between LTPA and mortality showed significantly higher all-cause, CVD, cancer and other cause mortality for the low LTPA group throughout the multiple analyses. Regarding desire to increase LTPA, both the “Yes, but I need support” and the “No” fractions within the low LTPA group had significantly increased ORs of all-cause mortality compared to the “Yes, and I can do it myself” reference fraction. No significant associations were observed for CVD mortality with regard to desire to increase physical activity in the low LTPA group. The no desire to increase LTPA fraction displayed significantly higher cancer mortality, and the “need support” fraction higher other cause mortality. Physical activity promotion measures to increase LTPA are particularly warranted in the low LTPA group, and the healthcare system and other contact surfaces for physical activity promotion should be considered.

## Ethical approval

This study was approved by the Ethical Committee (Etikprövningsnämnden) in Lund (No. 2010/343).

## Declaration of Competing Interest

The authors declare that they have no known competing financial interests or personal relationships that could have appeared to influence the work reported in this paper.

## Data Availability

The authors do not have permission to share data.
