# Supplementary material for: Leisure-time physical activity, desire to increase physical activity, and mortality: A population-based prospective cohort study
Source: Prev Med Rep. 2023 Apr 23;33:102212. doi: 10.1016/j.pmedr.2023.102212 (PMC10201835; doi:10.1016/j.pmedr.2023.102212)
Supplement: Supplementary data 1 [file mmc1.docx]

| **Supplementary Table 1.** Hazard rate ratios (HRRs) with 95% confidence intervals (95% CIs) of all-cause, cardiovascular (CVD), cancer and other cause mortality according to leisure-time physical activity (LTPA) in survival (Cox) regression models. The 2008 public health survey in Scania with 8.3 years follow-up.  Men and women combined. Total population **n=25 464**. **Weighted prevalence.** | | | | | | | | | |
| --- | --- | --- | --- | --- | --- | --- | --- | --- | --- |
|  | **Model 0** | | **Model 1** | | **Model 2** | | **Model 3** | |  |
| **Cause of death** | **HR** | **(95%CI)** | **HR** | **(95%CI)** | **HR** | **(95%CI)** | **HR** | **(95%CI)** | **Number of**  **deaths** |
| **All-cause mortality** |  |  |  |  |  |  |  |  | **1365** |
| Regular exercise | 1.0 |  | 1.0 |  | 1.0 |  | 1.0 |  |  |
| Moderate regular exercise | 0.8 | (0.7-1.1) | 0.9 | (0.7-1.1) | 0.9 | (0.7-1.1) | 0.9 | (0.7-1.1) |  |
| Moderate exercise | **1.8***** | (1.4-2.2) | 1.1 | (0.9-1.4) | 1.1 | (0.9-1.4) | 1.1 | (0.9-1.3) |  |
| Low LTPA | **3.8***** | (3.1-4.7) | **3.1***** | (2.5-3.8) | **2.6***** | (2.1-3.2) | **2.3***** | (1.8-2.9) |  |
| **Cardiovascular disease** |  |  |  |  |  |  |  |  | **416** |
| Regular exercise | 1.0 |  | 1.0 |  | 1.0 |  | 1.0 |  |  |
| Moderate regular exercise | 0.8 | (0.5-1.2) | 0.8 | (0.5-1.3) | 0.8 | (0.5-1.3) | 0.8 | (0.5-1.2) |  |
| Moderate exercise | **1.6*** | (1.1-2.3) | 1.0 | (0.7-1.5) | 1.0 | (0.7-1.4) | 0.9 | (0.6-1.3) |  |
| Low LTPA | **3.6***** | (2.5-5.3) | **3.0***** | (2.0-4.4) | **2.5***** | (1.6-3.7) | **1.9**** | (1.3-3.0) |  |
| **Cancer** |  |  |  |  |  |  |  |  | **528** |
| Regular exercise | 1.0 |  | 1.0 |  | 1.0 |  | 1.0 |  |  |
| Moderate regular exercise | 0.9 | (0.6-1.4) | 0.9 | (0.6-1.4) | 0.9 | (0.6-1.4) | 0.9 | (0.6-1.3) |  |
| Moderate exercise | **2.0***** | (1.4-2.9) | 1.3 | (0.9-1.8) | 1.3 | (0.9-1.8) | 1.2 | (0.9-1.7) |  |
| Low LTPA | **2.9***** | (2.0-4.3) | **2.3***** | (1.6-3.4) | **2.1***** | (1.4-3.0) | **1.9***** | (1.4-2.8) |  |
| **Others** |  |  |  |  |  |  |  |  | **421** |
| Regular exercise | 1.0 |  | 1.0 |  | 1.0 |  | 1.0 |  |  |
| Moderate regular exercise | 0.8 | (0.5-1.3) | 0.9 | (0.5-1.4) | 0.9 | (0.5-1.4) | 0.9 | (0.6-1.4) |  |
| Moderate exercise | **1.7**** | (1.2-2.5) | 1.1 | (0.7-1.6) | 1.1 | (0.7-1.5) | 1.0 | (0.7-1.5) |  |
| Low LTPA | **5.1***** | (3.5-7.5) | **4.1***** | (2.8-6.0) | **3.3***** | (2.3-4.9) | **3.2**** | (2.1-4.8) |  |
| Model 0 unadjusted. Model 1 adjusted for sex and age. Model 2 additionally adjusted for socioeconomic status (SES), country of birth and chronic disease. Model 3 additionally adjusted for BMI, daily smoking and alcohol consumption. | | | | | | | | | |
| Significance levels: * p<0.05, ** p<0.01, *** p<0.001; Weighted Hazard Ratios. Bootstrap method (1000 replicates) for variation estimation. | | | | | | | | | |

| **Supplementary Table 2.** Hazard rate ratios (HRRs) with 95% confidence intervals (95% CIs) of all-cause, cardiovascular (CVD), cancer and both cause mortality according to desire to increase physical activity in the low LTPA group in survival (Cox) regression models. The 2008 public health survey in Scania with 8.3 years follow-up.  Men and women combined. Total population **n=3 296^£^**. **Weighted prevalence.** | | | | | | | | | |
| --- | --- | --- | --- | --- | --- | --- | --- | --- | --- |
|  | **Model 0** | | **Model 1** | | **Model 2** | | **Model 3** | |  |
| **Cause of death** | **HR** | **(95%CI)** | **HR** | **(95%CI)** | **HR** | **(95%CI)** | **HR** | **(95%CI)** | **Number of**  **deaths** |
| **All-cause mortality** |  |  |  |  |  |  |  |  | **397** |
| “Yes, and I can do it myself” | 1.0 |  | 1.0 |  | 1.0 |  | 1.0 |  |  |
| “Yes, but I need support” | **2.3***** | (1.7-3.1) | **1.7***** | (1.3-2.3) | **1.4*** | (1.0-1.9) | **1.4*** | (1.0-2.0) |  |
| “No” | **3.5***** | (2.5-5.0) | **1.7**** | (1.2-2.4) | **1.5*** | (1.1-2.1) | **1.5*** | (1.1-2.1) |  |
| **CVD mortality** |  |  |  |  |  |  |  |  | **128** |
| “Yes, and I can do it myself” | 1.0 |  | 1.0 |  | 1.0 |  | 1.0 |  |  |
| “Yes, but I need support” | 1.5 | (0.9-2.6) | 1.1 | (0.7-1.9) | 1.0 | (0.5-1.7) | 1.0 | (0.6-1.7) |  |
| “No” | **2.8***** | (1.5-5.0) | 1.3 | (0.7-2.4) | 1.2 | (0.6-2.2) | 1.2 | (0.7-2.2) |  |
| **Cancer mortality** |  |  |  |  |  |  |  |  | **110** |
| “Yes, and I can do it myself” | 1.0 |  | 1.0 |  | 1.0 |  | 1.0 |  |  |
| “Yes, but I need support” | **1.9*** | (1.1-3.4) | 1.5 | (0.8-2.7) | 1.2 | (0.6-2.1) | 1.2 | (0.7-2.1) |  |
| “No” | **4.1***** | (2.2-7.6) | **2.2*** | (1.2-4.2) | **1.9*** | (1.0-3.6) | **2.0*** | (1.0-3.7) |  |
| **Other cause mortality** |  |  |  |  |  |  |  |  | **159** |
| “Yes, and I can do it myself” | 1.0 |  | 1.0 |  | 1.0 |  | 1.0 |  |  |
| “Yes, but I need support” | **3.8***** | (2.1-6.8) | **2.8***** | (1.5-5.0) | **2.3**** | (1.2-4.2) | **2.2*** | (1.1-4.3) |  |
| “No” | **4.1***** | (2.2-7.6) | **1.9*** | (1.1-3.4) | 1.7 | (1.0-3.0) | 1.6 | (0.9-2.9) |  |
| Model 0 unadjusted. Model 1 adjusted for sex and age. Model 2 additionally adjusted for socioeconomic status (SES), country of birth and chronic disease. Model 3 additionally adjusted for BMI, daily smoking and alcohol consumption. **^£^**These observations belong to group low LTPA in the variable leisure-time physical activity. | | | | | | | | | |
| Significance levels: * p<0.05, ** p<0.01, *** p<0.001; Weighted Hazard Ratios. Bootstrap method (1000 replicates) for variation estimation. | | | | | | | | | |

| **Supplementary Table 3.** Odds ratios (ORs) with 95% confidence intervals (95% CIs) of all-cause, cardiovascular (CVD), cancer and other cause mortality according to leisure-time physical activity (LTPA) in logistic regression models. The 2008 public health survey in Scania with 8.3 years follow-up.  Men and women combined. Total population **n=25 464**. **Weighted prevalence.** | | | | | | | | | |
| --- | --- | --- | --- | --- | --- | --- | --- | --- | --- |
|  | **Model 0** | | **Model 1** | | **Model 2** | | **Model 3** | |  |
| **Cause of death** | **OR** | **(95%CI)** | **OR** | **(95%CI)** | **OR** | **(95%CI)** | **OR** | **(95%CI)** | **Number of**  **deaths** |
| **All cause mortality** |  |  |  |  |  |  |  |  | **1365** |
| Regular exercise | 1.0 |  | 1.0 |  | 1.0 |  | 1.0 |  |  |
| Moderate exercise | **1.5***** | (1.2-1.8) | 1.1 | (0.9-1.3) | 1.0 | (0.8-1.3) | 1.0 | (0.8-1.3) |  |
| Low LTPA | **3.9***** | (3.2-4.9) | **3.5***** | (2.8-4.5) | **2.9***** | (2.3-3.7) | **2.5***** | (2.0-3.3) |  |
| **CVD mortality** |  |  |  |  |  |  |  |  | **416** |
| Regular exercise | 1.0 |  | 1.0 |  | 1.0 |  | 1.0 |  |  |
| Moderate exercise | 1.3 | (0.9-1.3) | 1.0 | (0.7-1.4) | 0.9 | (0.6-1.4) | 0.8 | (0.6-1.2) |  |
| Low LTPA | **3.6***** | (2.4-5.3) | **2.7***** | (1.8-4.2) | **2.2***** | (1.5-3.4) | **1.7*** | (1.1-2.7) |  |
| **Cancer mortality** |  |  |  |  |  |  |  |  | **528** |
| Regular exercise | 1.0 |  | 1.0 |  | 1.0 |  | 1.0 |  |  |
| Moderate exercise | **1.6**** | (1.2-2.3) | 1.2 | (0.9-1.7) | 1.2 | (0.8-1.7) | 1.1 | (0.8-1.6) |  |
| Low LTPA | **2.9***** | (2.0-4.2) | **2.1***** | (1.4-3.1) | **1.9***** | (1.3-2.8) | **1.7**** | (1.1-2.5) |  |
| **Other cause mortality** |  |  |  |  |  |  |  |  | **421** |
| Regular exercise | 1.0 |  | 1.0 |  | 1.0 |  | 1.0 |  |  |
| Moderate exercise | 1.4 | (0.9-2.1) | 1.0 | (0.7-1.5) | 1.0 | (0.7-1.5) | 1.0 | (0.7-1.5) |  |
| Low LTPA | **5.0***** | (3.4-7.5) | **3.9***** | (2.6-5.9) | **3.1***** | (2.0-4.8) | **2.9***** | (1.9-4.7) |  |
| Model 0 unadjusted. Model 1 adjusted for sex and age. Model 2 additionally adjusted for socioeconomic status, country of birth and chronic disease. Model 3 additionally adjusted for BMI, daily smoking and alcohol consumption. Significance levels: * p<0.05, ** p<0.01, *** p<0.001; Weighted Hazard Ratios. Bootstrap method (1000 replicates) for variation estimation. | | | | | | | | | |
